# Hospital-Related Determinants of Refusal of Organ Donation in France: A Multilevel Study

**DOI:** 10.3390/ijerph22040618

**Published:** 2025-04-15

**Authors:** Régis Bronchard, Gaëlle Santin, Camille Legeai, Anne Bianchi, Séverine Grelier, Caroline Bogue, Olivier Bastien, François Kerbaul

**Affiliations:** 1 Direction Prélèvement Greffe Organes-Tissus, Agence de la Biomédecine, 93212 Saint-Denis la Plaine, France; gaelle.santin@biomedecine.fr (G.S.); anne.bianchi@biomedecine.fr (A.B.); olivier.bastien@biomedecine.fr (O.B.); francois.kerbaul@biomedecine.fr (F.K.); 2 Pôle Sécurité Qualité, Agence de la Biomédecine, 93212 Saint-Denis la Plaine, France; severine.grelier@biomedecine.fr; 3 Pôle Recherche Europe International et Veille, Agence de la Biomédecine, 93212 Saint-Denis la Plaine, France; caroline.bogue@biomedecine.fr

**Keywords:** donation after brain death, refusal, hospital-related determinants, organ donation process audit, multilevel analysis

## Abstract

In a worldwide context of organ shortage, refusal of organ donation remains the main reason for the non-procurement of organs from deceased donors. Many studies have characterized the clinical or psychological factors of refusal but not organizational and structural factors in healthcare centers. We used multilevel logistic regression models with hospitals as a random effect to analyze organ procurement among 6734 potential brain-dead donors recorded in the national register in France in 2018 and 2019. According to the opt-out law, 29% of the potential donors refused to donate. Among hospital-related factors, low probability of refusal was related to hospitals audited for the organ donation process by the national program (adjusted odds ratio [aOR] 0.74, 95% confidence interval [CI]: 0.58–0.94), hospitals with high inpatient satisfaction scores for care (aOR 0.95, 95% CI 0.92–0.99) and facilities with a high ratio of nurse donor coordinators to donors (aOR: 0.78; 95% CI: 0.64–0.95). Among clinical factors, the odds of refusal were associated with age younger than 65 years (18–49 years; aOR 1.58, 95% CI 1.37–1.83) and donors with blood group B (aOR 1.32, 95% CI: 1.10–1.59). Hospital-related factors are just as important as individual factors in the procurement of organs from potential brain-dead donors.

## 1. Introduction

For some patients, organ transplantation is the only possible treatment. Worldwide, despite an increase in number of organs procured over the last decade, the volume available does not meet current needs. Accordingly, wait times for organs are continuously increasing, and many patients die while on the wait list.

In 2023, France ranked 10th worldwide and 9th in Europe in terms of actual per-million population [pmp] deceased donors (27.6 vs. 49.4 pmp in Spain and 48.0 pmp in the United States). It ranked sixth worldwide and fourth in Europe in terms of pmp transplant patients (84.1 vs. 132.1 pmp in the United States and 120.4 pmp in Spain) [1]. In France, there were 8716 new registrants on the waiting list and 5633 transplantations, which resulted in an increase in the waiting list to 11,435 patients registered on the active waiting list at the beginning of 2024 and an increased median wait time (28.4 months for kidney transplantation) as well as 869 deaths on the wait list [2]. Most grafts came from deceased donors (27.6 actual deceased donors pmp, mostly donation after brain death [DBD], 4.3 donation after cardiac death [DCD] pmp), and also from living donors (8.6 living donation pmp) [1].

Therefore, actions are needed to increase the pool of donors. In France, the aims of national policy are to find ways to increase living and deceased donation (both DBD and DCD donors) [3]. For DCD donors, the main strategy is to increase the number of centers involved in controlled DCD donation. For living donors, the main strategy is to work with each living donation center to adapt local strategies to improve living donation. For DBD donors, recommendations occur at several levels: upstream, by increasing the pool of potential donors by improving their identification or using donors with expanded criteria; and then, once potential donors have been identified, by limiting logistical and care failures for the donor or minimizing refusal of the procurement of organs [4,5]. In this respect, better care of donors and their families could be achieved with better hospital organization around organ and tissue donation. Such organization could include enhancing the organ donation process in healthcare centers, relying on hospital donor coordination teams with experienced and continuously trained staff, and monitoring all stages of the donation process by using quality indicators [6]. In France, as in many other countries, although organ donation is a statutory public health priority with a reinforced opt-out law, refusal is the principal cause of non-procurement each year [2]. Successful actions to reduce this rate require understanding its determinants.

Many studies have demonstrated that clinical factors, factors related to the quality of the donor’s care, and donor socioeconomic and cultural factors all affect refusal rates [6,7,8,9,10,11,12,13]. However, no quantitative study has considered organizational factors at the level of the hospitals involved in caring for potential organ donors.

This study aimed to quantify the weight of organizational and structural criteria in the refusal of organ donation by using multilevel logistic regression to take into account the hierarchical structure of the data and individual factors already documented in the literature.

## 2. Materials and Methods

### 2.1. Management of Organ Donation in France

In France, the recovery and transplantation of organs and tissue are supervised by the Agence de la biomedecine (ABM), a national public establishment that reports to the Ministry of Health. The ABM’s mission is to manage the refusal register; coordinate the procurement of organs and their anonymization, distribution, and attribution; ensure the evaluation of the medical activities it oversees; and support healthcare professionals.

In France, 165 hospitals are authorized for organ procurement around the country. The hospitals so authorized are the larger hospitals in each region of France and overseas; they are the ones able to provide and perform the donation process and form a proximity network with smaller hospitals in their area to detect potential donors in every hospital and all areas in France. The authorized hospitals have a hospital donor coordinating team (DCT) for recovering organs and tissues. This multiprofessional team consisting of physicians and nurses coordinates all stages of organ procurement from donors. Once a hospital department has detected a possible donor [14], the DCT assesses the possibility of procurement and interviews the potential donor’s family (and/or close friends or relatives, as appropriate; hereafter “the family”). In France, as in other European countries that apply the principle of presumed consent [6,15,16], the law provides that unless an adult has affirmed while alive that they do not want their organs donated, every adult person who dies is a potential donor [17,18]. A “refusal register” allows individuals to express their refusal. Several other methods are available, such as informing a family member or close friend, orally or in writing. DCTs can also decide to stop the organ donation procedure in various contexts [17,18]. If no refusal is reported, the DCT coordinates the procurement management (data collection, support for the local teams and the family), in direct contact with the ABM after it has opened the donor file (via CRISTAL, the ABM’s secure application).

The ABM supports healthcare facilities throughout France on a daily basis, including DCTs, in developing their procurement activities. It also has reliable information about the DCT staff in each facility. Furthermore, the ABM regularly conducts audits of the hospitals authorized to procure organs and tissues by using a national self-assessment tool prospectively completed by the hospital and evaluated by the auditors before a 2- or 3-day visit of the hospital and the auditors meeting with teams to evaluate each item of the process. The audit’s assessment of the organ procurement process analyses the strategy, available human resources and logistics, education, communication, operational process for identifying potential donors, diagnosis, care of donors and the family, interview process of families, organization of organ and tissue procurement, quality process—risk management and biovigilance. The recommendations formulated in the final report are integrated into an action plan to improve the quality and safety of the institution’s processes and serve as a roadmap to improve organ donation.

### 2.2. Population

The population for this study corresponded to all potential donors with brain death recorded in 2018 and 2019 (before the COVID-19 period) in France (metropolitan France and overseas departments, regions, and territories) who died in an establishment authorized to procure organs. We excluded potential donors whose families were not approached by the DCT as well as those from hospitals not eligible for the audits, that is, patients who died in an establishment not authorized to procure organs and who were not transferred to such a hospital.

### 2.3. Data Sources

#### 2.3.1. CRISTAL

The study population and most of the data were extracted from CRISTAL, the French national organ transplant register, managed by the ABM. It centralizes the data for deceased potential donors (whether or not their organs were actually procured or used). There are no data on ethnic origin in the Cristal database because French law forbids the collection of such data. French law defines research studies based on this national register as part of the evaluation of transplantation and therefore not requiring additional approval by an institutional review board. The database has been reported to the national commission for information technology and privacy (CNIL).

To obtain complementary data about hospitals, the CRISTAL database was linked to several external databases by the hospital identifier attributed to it by the French state.

#### 2.3.2. ABM Databases Monitoring Hospitals and DCT Staff

Internal ABM audit databases and the number of DCT staff (nurses and physicians) were linked to CRISTAL data.

#### 2.3.3. External ABM Databases

Annual Statistics of Health-Care Facilities [19] made available by the Ministry of Health’s and Quality indicators for hospitals [20,21] made available by the French national authority for health (Haute autorité de santé) were also used.

- Annual Statistics of Health-Care Facilities [19]

This database results from the Ministry of Health’s mandatory, exhaustive, annual administrative survey of French hospitals. It produces data on each establishment’s specific characteristics: structure, departments, capacity, technical platforms equipment, and staff.

- Quality indicators for hospitals
  a. Hospital certification

Hospital certification is an external system for evaluating the quality and safety of care in France [20]. These certifications, mandatory for all hospitals, are conducted every 4 years by professionals assigned by the French national authority for health (Haute autorité de santé [HAS]), an independent public authority. In 2018–2019, the data collected for certification focused on 28 areas covering themes such as contact with patients’ families, patient care, and organization of the operating theater.

    b. An indicator of care quality and safety: e-Satis

The adjusted patient satisfaction (e-Satis) score ranges from 0 to 100. This indicator is the national gauge used for the continuous measurement of patient satisfaction and experience [21]. The score, based on HAS-validated patient questionnaires, has been used since April 2016 in all hospitals in France whether they are authorized for organ procurement or not. The HAS-validated questionnaires cover dimensions of the important stages of patient healthcare pathways, including their reception and care by physicians and surgeons as well as by nurses and nurses’ aides. Adjustment was based on two variables included in the patient questionnaire: satisfaction with life in general and improvement in health status following a stay in the establishment.

### 2.4. Variable of Interest

We considered two types of refusal of organ procurement for donation [2,18]: the deceased’s refusal to donate while alive and refusal resulting from a “context that finally did not result in organ recovery” after excluding refusal to donate by the deceased’s legal guardian or representative for minors (age 0–17 years) and those legally unable to consent. The potential donor may have directly expressed this refusal while alive in a written document sent to the refusal register or left with family members or stated it to a family member. The “context” in which procurement did not occur corresponds to several situations assessed by DCTs: refusal when the family does not understand brain death or when a clear statement of the patient’s position on donation while alive cannot be obtained for cultural reasons or because of conflict within the family or the healthcare team.

### 2.5. Exposure Factors

To study refusal, we examined three sets of factors, related to the donor, the hospital, and the DCT group (which may cover several hospitals in the network). The analyses were adjusted for age, sex, blood group, cause of death, and suicide as the context of death.

The hospital variables studied were its location (mainland France or overseas territories and regions), type of hospital (university hospital center or public general hospital), number of beds, and presence of a neurovascular unit. We also studied the hospital’s HAS certification as well as its e-Satis scores: inpatient satisfaction score, outpatient satisfaction score, satisfaction score for reception, satisfaction score for care by nurses and nurses’ aides, and satisfaction score for care by physicians and surgeons. We also assessed whether the ABM had already audited the hospital’s donation process.

Finally, we studied the following indicators related to the DCT: mean number of full-time equivalent (FTE) nurses in each DCT in 2018 and 2019, mean proportion of new DCT staff (<1-year seniority), mean number of FTE physicians and their turnover (at least one physician with <1-year seniority), staff shifts of after-hours offsite on-call duty, difference between number of FTE physicians or nurses recommended by the ABM and that observed, ratio of number of FTE physicians to number of potential donors, and ratio of number of FTE DCT nurses to number of potential donors. The ratios were classified according to the median estimated in our data.

### 2.6. Statistical Analysis

Refusal of organ donation was analyzed by characteristics of donors and hospitals by chi-squared tests for categorical variables and Student t test for continuous variables, with significance defined as *p* < 0.05. Because patients in a given hospital share characteristics associated with it, the data present a hierarchical structure, so standard statistical analyses are inappropriate. For this reason, we used multilevel logistic regression with the hospital as a random effect following the recommendations of Austin and Merlo [22]. We estimated adjusted odds ratios (aORs) and 95% confidence intervals (CIs). The final model was constructed in three steps:

- Step 1: empty model (i.e., a model with no exposure factor) with a random intercept, enabling us to estimate the hospital-related effect without taking particular individual or contextual factors into account. A significant random effect found with this model points in the direction of a crude difference in refusal rates between hospitals. However, if the result is not significant, there is no point in using multilevel models thereafter because the assumption of a hierarchical structure of the data is not verified.
- Step 2: model with a random intercept and donor characteristics. Donor characteristics associated with refusal of organ donation at *p* < 20% on univariable analysis were included in a backward stepwise logistic regression. Variables associated with refusal at a threshold of 5% were kept in the final step 2 model. This model allows for taking into account clinical variables that may be indirectly linked to the hospital. A significant random effect found with the final step 2 model points in the direction of a structural difference in refusal rates between hospitals, in other words there is a hospital effect not explained by the individual characteristics of donors. However, if the result is not significant, there is no point in using multilevel models thereafter because the assumption of a hierarchical structure of the data is no longer verified once the variables relating to the donor have been taken into account. Hence, donor variables must be included in an intermediate analysis so as not to wrongly conclude that there is a structural effect of the hospital in the refusal to donation.
- Step 3: final model with a random intercept and characteristics of both donors and hospitals. Hospital characteristics associated with refusal of organ donation at *p* < 20% on univariable analysis were included in a backward stepwise logistic regression with donor characteristics selected in the final step 2 as fixed factors. Variables associated with refusal at a threshold of 5% were kept in the final model. Furthermore, if the *p*-value associated with the random effect disappeared, this means that the hospital effect is fully explained by the donor and hospital factors included in the final model.

The random intercept, with its associated variance, allows for testing a difference between hospitals regarding refusal to organ donation for each model. The variance estimates of the random intercepts can also be used to measure the contribution of the “donor” variables in the step 2 final model and the “hospital” variables in step 3 final model. Two statistical indicators are available for this purpose (1) PCV (Proportional change of variance) = 100 × (Variance 1 − Variancei)/Variance1, where 1 = model 1 (empty model) and i = 2 for the random intercept model with donor variables and i = 3 for the random intercept model with donor and hospital variables. Therefore, the PCV can be used to quantify the reduction in variance after adjustment for the donor variables and after adjustment for the hospital variables, thus the importance of the studied factors to explain the refusal to donation. (2) The VPC(ICC) measures the degree of similarity between donors in the same group. If a VPC(ICC) remains in the final model, it means that it remains an inter-individual variability linked to the hospital, so there is still an unexplained hospital effect.

### 2.7. Sensitivity Analysis

Sensitivity analysis was performed to assess the consistency of our results: we analyzed the context that did not finally result in organ donation, after excluding minors and adults under guardianship. In addition, we excluded potential donors who refused donation by registering with the refusal register or in a written document during their lifetime so that we could focus on the post-mortem refusals.

All analyses were conducted with SAS Enterprise Guide version 8.3 (SAS Institute, Cary, NC, USA).

## 3. Results

The CRISTAL database contained data for 6955 potential brain-dead donors in France in 2018 and 2019. The study excluded 21 donors whose families were not interviewed about their views on donation and the 200 donors who died in a hospital not authorized to recover organs, thus potential donors but not transferred to a procurement hospital (most often because of refusal: 79.5%). These eliminations allowed for complete hospital information because the audit information was available from only hospitals authorized to procure organs. The analysis finally included 168 hospitals (6734 potential donors).

### 3.1. Description of Refusal Rate

Of the 6734 potential donors included in the study, 1956 (29%) were classified as refusals: 48.8% had expressed their refusal while alive, reported in writing after brain death by a family member; legal guardians or representatives refused for 5.3%; and 1.1% were registered in the refusal register. For 954 potential donors (44.8%), the context did not finally result in organ recovery (Table 1).

The refusal rate (Table S1) decreased with increasing age: it was 41.3% for minors, 32.9% for patients aged 18 to 49 years, 30.9% for those aged 50 to 64 years, and 24.5% for those aged ≥65 years. It was higher for donors with blood group B (33.6% vs. <29% for the other blood groups). It was lower for those who died in a context of suicide (24.9% vs. 29.3% for the other types of deaths; *p* = 0.05). For hospital characteristics, the refusal rate (Table S1) was higher in UHCs than non-UHCs (31.1% vs. 26%), fewer number of beds (32% vs. less than 31% for other hospital sizes) and reduced adjusted satisfaction scores for reception, outpatient and inpatient satisfaction and care by nurses/nurses’ aides. Refusal was associated with increased mean proportion of new DCT nurses, <3 FTE DCT physicians/100 potential donors and <8 FTE DCT nurses/potential donors, increased difference between FTE physicians recommended to that observed and <2 mean number of after-hours offsite on-call duty shifts.

### 3.2. Refusal of Organ Donation After Adjustment for the Hospital-Related Random Effects

**Figure 1:**
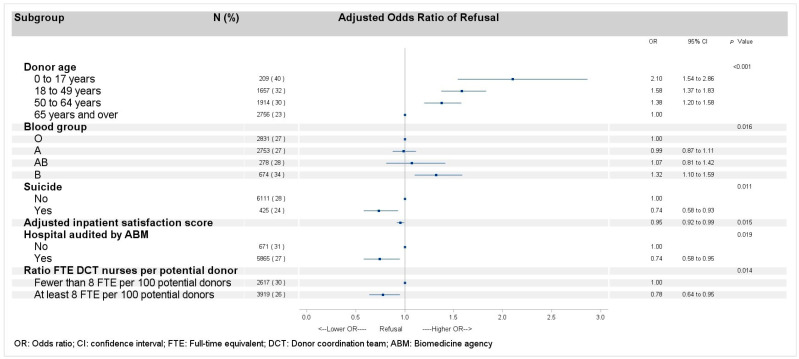
Multilevel logistic regression model of factors related to refusal.

High odds of donation refusal (Figure 1, Table S1) were associated with potential donors belonging to the age groups 0 to 17 years (aOR 2.10, 95% CI 1.54–2.86), 18 to 49 years (aOR 1.58, 95% CI 1.37–1.83), and 50 to 64 years (aOR 1.38, 95% CI 1.20–1.58) versus ≥65 years (*p* < 0.0001). It was associated with donors with blood group B (aOR 1.32, 95% CI 1.10–1.59; *p* = 0.02) versus blood group O. Low odds of refusal was associated with death in a context of suicide (aOR 0.74, 95% CI 0.58–0.93; *p* = 0.01).

For hospital-related factors, low odds of refusal were associated with inpatient satisfaction score (aOR 0.95, 95% CI 0.92–0.99; *p* = 0.02) and hospitals that had been audited by the ABM (aOR 0.74, 95% CI 0.58–0.95; *p* = 0.02) as well as hospitals with a ratio of FTE DCT nurses to donors ≥8 (aOR 0.78, 95% CI 0.64–0.95; *p* = 0.01).

Regardless of the model (Table 2), the variance of the random effects was significant. Therefore, refusal of organ donation differed among hospitals, even after taking into account the adjustment factors linked both to donors and hospitals. The proportion of variance of the random hospital-related effect explained by the models was 12.2% after adjustment for donor factors and 25.2% after adjustment for both donor and hospital factors. Therefore, hospital-related factors may be as important as donor-related factors in the refusal of donation because the PCV for model 3 was twice as large as the PCV for model 2. The proportion of total individual variation attributable to variations between hospitals [VPC(ICC)] was 5.9% in the empty model (model 1) and 4.6% after taking into account donor and hospital factors. Thus, 4.6% of the total inter-individual variability was linked to the hospital, so there is still an unexplained hospital effect. Furthermore, although refusal rates were significantly higher in UHCs than non-UHCs on univariate analysis, this difference disappeared after adjustment on donor age, donors in UHCs being younger than donors in non-UHCs.

These two indicators showed that hospital-related factors carry as much weight as individual factors in the association with donation refusal.

### 3.3. Sensitivity Analysis

**Figure 2:**
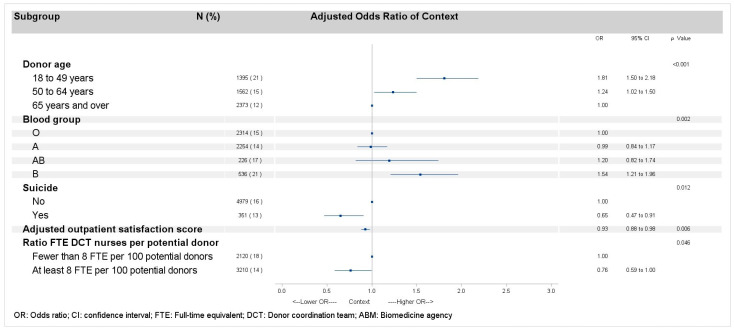
Multilevel logistic regression models of factors related to context, after excluding all types of refusals for deceased donors, minors, and protected adults.

Factors associated with “a context that did not finally result in organ recovery” were globally the same as those associated with refusal (Figure 2). The only hospital-related factor not associated with context was the hospital having been audited, but nonetheless, it remained at the borderline of significance (*p* < 0.10).

## 4. Discussion

This multilevel multivariate analysis of a large cohort of potential brain-dead donors performed during a 2-year recent period identified potential hospital-related determinants of refusal of organ donation. The rate of organ donation refusal was reduced with an adequate workforce of DCT nurses, an audit of hospitals for their procurement management and donation process, and better inpatient satisfaction. Hospital-related factors carried as much weight as individual factors in the association with donation refusal. Moreover, the odds of refusal were decreased with increasing donor age and with death in connection with suicide but was increased for donors with blood group B.

In France, as generally throughout the world, refusal of organ donation is the leading cause of non-procurement of organs from potential donors with brain death [2,6]. The refusal rate for organ donation in France has been oscillating between 29% and 36% for more than 20 years [2]. Few quantitative studies are available on organizational factors associated with refusal; indeed, many differences exist between countries on procurement legislation and procurement management [4,6,23,24]. Furthermore, the literature on organizational factors related to procurement rarely focuses on donation at the hospital level but usually at a national funding resources level [23]; one of the advantages of this study is that it highlights these factors specifically associated with refusal and quantifies them.

This study pointed to the importance of the hospital DCT team in limiting donation refusal. The odds of donation refusal were decreased with an adequate number of DCT nurses on the hospital DCT team. The same result was found for DCT physicians but only on univariable analysis. In France, DCT nurses spend the most time with donor families; an adequate number of DCT nurses means that time can be taken to discuss organ donation with relatives at different times [5,25,26]. Several studies emphasize the key role of the DCT (nurses or physicians) as a cornerstone of care of the donor’s family, accompanying them during and after the donation process [5,25], but also as dedicated professionals enhancing the organ donation culture in the whole healthcare center and facilities as well as outside the hospital (providing education and communication) [5,6,7,8,11,12,25,26,27,28,29,30].

A qualitative study of DCT nurses in Spain showed that they play a fundamental role in increasing consent to donation [26]. It also emphasized that dealing with death and the grief of loved ones can be a heavy burden; having the right number of staff certainly would help colleagues support each other. In addition, a great many skills are required (knowledge of the organization of organ donation, exchanges with the teams involved at the hospital and with relatives) in a limited time, so if there are enough nurses, they will probably have more time to carry out all their tasks. In our study, seniority, assessed by physician turnover and the proportion of new nurses in the DCT team, was not associated with refusal to donate. This finding does not agree with the publication on DCT nurses [26]; it may be related to the proxy indicators we used because we could not create fine-tuned seniority categories with the quality of our data or to our comparison with a qualitative study.

The second finding about organizational factors is the low odds of refusal associated with donor hospitals that had their organ donation process audited by the ABM. In France, audits are carried out at hospital level to take into account the entire procurement chain. They are carried out in close collaboration with the hospital teams and over the long term, enabling them to reflect on how they operate and to better identify areas for improvement, with the necessary buy-in to ensure that the audits are followed up with effective action plans. The importance of audits in the organization of procurement have been reported in several publications [5,6,12,31,32,33]. Our study shows that auditing is also important in reducing donation refusal. The link between audits and low odds of refusal suggests not only that they improve reception and communication with relatives but also that consent to donation is part of a more global approach.

This recommendation should be viewed in light of our finding of low odds of refusal to donate associated with good inpatient satisfaction with hospital care [20,21]. Although this result is similar to that from several qualitative sociological studies, this is the first quantitative result in this area. This satisfaction score takes into account reception, care, accommodation and the organization of discharge [21]. This multifactorial indicator of the quality of care from the patient’s point of view, combined with donation refusal, supports the published recommendations, which advocate effective working relationships between stakeholders at all stages [5] and the importance of relatives’ trust in the hospital system for consent to donation [25].

We also found clinical factors associated with low odds of refusal. Probability of refusal decreased with increasing patient age and was lowest for those aged ≥65 years. This finding contrasts with some reports in the literature from France and elsewhere, finding the highest refusal rates among both the youngest and oldest age groups [7,8,10,34,35]. For the oldest group of donors, this finding may signal a bias, specifically, the progressive development in France of intensive care to facilitate organ donation [14,36,37,38], particularly in the oldest potential donors. The highest opposition rate was seen in donors < 18 years old for whom French law requires written consent from both parents to proceed to organ donation.

High probability of refusal was associated with blood group B. Group B could be an indirect indicator of minority ethnicity of the donor, which is a known risk factor of higher refusal rate [7,10,13]. Indeed, an high proportion of people from Africa and Asia, which are minority ethnicities in France, have blood group B [39,40,41]. Because collecting data on ethnic origin is forbidden by law in France, the CRISTAL database does not collect such data, so we cannot confirm our hypothesis. Moreover, the link between minority ethnicity status and increased refusal rate [7,10,11,13,42] could be a reflect of lower socio-economic levels of minority ethnicities because in France, the socioeconomic level of donors is, in part, related to their migration origin [43]. These factors are also risk factors of increased refusal rates, but we have no data on socioeconomic level of donors in our database.

Cause of death was not associated with refusal, but a context of suicide was associated with low probability of refusal, as previously reported [34]. The context of death after suicide may be less conducive to refusals but should be confirmed.

The major strength of our study is its inclusion of hospital-related organizational variables. The study of the variance of random effects shows the importance of considering hospital-related factors in the refusal to donate organs. Furthermore, this work presents the organization of organ donation in France, which will help to build up the literature for future research, in that many studies provide comparisons between countries and France has been absent until now due to a lack of published articles [4,5,31,44].

Our study has some limitations. The opt-out policy in France may limit the extrapolation of these results to countries requiring explicit consent to donate. Nevertheless, a systematic review of the literature did not find a significant link between national policies about refusal [42]. Another limitation stems from the exclusion of some potential brain-dead donors in hospitals that were not authorized to procure organs and not transferred to authorized hospitals because of lack of a DCT at those sites and their non-inclusion in ABM audits did not allow for studying their hospital data. Nevertheless, the bias in our results is probably negligible because the authorized hospitals have equivalent hospital networks. However, this result should encourage DCTs to work on detection and donor and family care and to reinforce audits in such hospitals.

## 5. Conclusions

Refusal remained the principal cause of the non-donation of organs from potential brain-dead donors in France in 2018 and 2019. Our results suggest that DCTs, as the cornerstone of organ donation, must be sufficiently staffed and that periodic audits of hospitals involved in organ procurement should be mandatory and reinforced. These concrete actions are ways to reduce refusal of organ donation, helping to improve the donation culture throughout the hospital, which includes better care for patients and their families. An environment of enhanced satisfaction is conducive to organ donation, which in turn allows for developing a relationship of trust between patients and their families and the hospital system, trust being a major factor in limiting the refusal of organ donation. Although the refusal rate was about 30% before the COVID-19 pandemic, it increased to 33% between 2020 and 2022, reaching 36% in 2023 and 2024. Given the impact of the pandemic on hospitals in France, particularly in terms of hospital staff resignations leading to reductions in the number of intensive care beds, our study could be repeated in the future to investigate new organizational factors potentially associated with an increase in organ donation refusal rates.

## Figures and Tables

**Table 1:** Description of the components of refusal of organ and tissue recovery.

	N	%
Context that finally did not result in organ procurement	876	44.8
Refusal by deceased while alive (refusal register or written refusal)	22	1.1
Refusal by legal guardian or representative	104	5.3
Refusal expressed while alive, reported in writing by a family member * after interview with DCT	954	48.8

DCT: Donor coordinating team. * Family members include close friends or relatives.

**Table 2:** Components of the variance of multilevel logistic regression models.

	Empty Model	Model 2	Model 3
Variance random effects	0.2078 *	0.1824 *	0.1554 *
PCV	reference	12.2%	25.2%
VPC (ICC)	5.9%	5.3%	4.6%

PCV: proportional change of variance; VPC: variance partition coefficient; ICC: intraclass correlation. * *p*-value < 0.001.

## Data Availability

Main data used in this study were extracted from the CRISTAL registry, coordinated and supported by the French Agence de la biomédecine. Access to national data is regulated by a scientific committee of the French Agence de la biomédecine, which analyzes each request. Thus, data cannot be made publicly available because of legal restrictions. Data are available upon request. If readers need information about the data from the CRISTAL registry, they can contact Nicolas Chatauret (nicolas.chatauret@biomedecine.fr). SAE data are available upon request in this website: https://data.drees.solidarites-sante.gouv.fr/pages/accueil/ (accessed on 22 August 2023). Annual Statistics of Health-Care Facilities Hospital certification are available at this website: https://www.data.gouv.fr/fr/datasets/certification-des-etablissements-de-sante-pour-la-qualite-des-soins-referentiel-2014-2020/ (accessed on 22 August 2023). An indicator of care quality and safety: e-Satis is available at this website: https://www.data.gouv.fr/fr/datasets/indicateurs-de-qualite-et-de-securite-des-soins-mesure-de-la-satisfaction-dispositif-e-satis-recueil-2018/ (accessed on 18 July 2023).

## Supplementary Materials

The following supporting information can be downloaded at: https://www.mdpi.com/article/10.3390/ijerph22040618/s1, Table S1: Refusal of organ donation by potential donor et hospital characteristics; Table S2: Multilevel logistic regression models of factors related to refusal of organ donation.

## Abbreviations

The following abbreviations are used in this manuscript:

ABM	Agence de la biomédecine
DCT	Donor coordination team
HAS	Haute autorité de santé (national authority for health)
FTE	Full-time equivalent
DBD	Donation after brain death
DCD	Donation after cardiac death
pmp	Per million population
