# Supplementary material for: Hospital-Related Determinants of Refusal of Organ Donation in France: A Multilevel Study
Source: Int J Environ Res Public Health. 2025 Apr 15;22(4):618. doi: 10.3390/ijerph22040618 (PMC12026945; doi:10.3390/ijerph22040618)
Supplement: Supplementary file 1 [file ijerph-22-00618-s001.zip › ijerph-3564600-supplementary.pdf]

Table S1. Refusal of organ donation by potential donor et hospital characteristics.

	N (%)	% Missing Data	No Refusal	Refusal	*P* value
**Potential donor characteristics**					
**Potential donors**	6734 (100%)	0	4778 (71.0)	1956 (29.0)	
**Donor age, years**					<0.01
0 to 17	223 (3)	0	131 (58.7)	92 (41.3)	
18 to 49	1712 (25)		1149 (67.1)	563 (32.9)	
50 to 64	1965 (29)		1358 (69.1)	607 (30.9)	
≥65	2834 (42)		2140 (75.5)	694 (24.5)	
**Donor sex**					0.27
Female	2952 (44)	0	2115 (71.6)	837 (28.4)	
Male	3782 (56)		2663 (70.4)	1119 (29.6)	
**Blood group**					<0.01
A	2766 (42)	2.4	2025 (73.2)	741 (26.8)	
AB	279 (4)		200 (71.7)	79 (28.3)	
B	681 (10)		452 (66.4)	229 (33.6)	
O	2849 (43)		2070 (72.7)	779 (27.3)	
**Cause of death**					0.06
Vascular	3824 (57)	0	2750 (71.9)	1074 (28.1)	
Trauma	1426 (21)		1021 (71.6)	405 (28.4)	
Anoxia	1303 (19)		883 (67.8)	420 (32.2)	
Poisoning	25 (0)		17 (68)	8 (32)	
Other	156 (2)		107 (68.6)	49 (31.4)	
**Suicide**					0.05
No	6261 (94)	0.6	4428 (70.7)	1833 (29.3)	
Yes	430 (6)		323 (75.1)	107 (24.9)	
**Hospital characteristics**					
**Hospital in overseas region or territory, n (%)**					
No	246 (4)	0	173 (70.3)	73 (29.7)	0.83
Yes	6488 (96)		4605 (71)	1883 (29)	
**Type of hospital, n (%)**					
UHC	3984 (59)	0	2743 (68.9)	1241 (31.1)	**<0.01**
Non-UHC	2750 (41)		2035 (74)	715 (26)	
**Number of beds of hospital site of death, n (%)**					
<200	441 (7)	0	300 (68)	141 (32)	**0.02**
200 to 299	1272 (19)		904 (71.1)	368 (28.9)	

300 to 399	1488 (22)		1074 (72.2)	414 (27.8)	
400 to 499	1248 (19)		921 (73.8)	327 (26.2)	
≥500	2285 (34)		1579 (69.1)	706 (30.9)	
**Neurovascular unit, n (%)**					
No	1430 (21)	0	1023 (71.5)	407 (28.5)	0.58
Yes	5304 (79)		3755 (70.8)	1549 (29.2)	
**Hospital audited by the ABM, n (%)**					
No	687 (10)	0	467 (68)	220 (32)	0.07
Yes	6047 (90)		4311 (71.3)	1736 (28.7)	
**HAS quality indicators, n (%)**					
**Level of certification**					
Certification with or without recommendations for improvement	6183 (92)	0	4389 (71)	1794 (29)	0.85
Certification with obligations to improve or decision suspended	551 (8)		389 (70.6)	162 (29.4)	
**Adjusted satisfaction score for reception, mean (SD)**	6734	0	70.8 (3.2)	70.6 (3.4)	**0.01**
**Adjusted outpatient satisfaction score, mean (SD)**	6734	0	78.2 (2.2)	77.8 (2.4)	**<0.01**
**Adjusted inpatient satisfaction score, mean (SD)**	6734	0	72.3 (2.2)	72.1 (2.3)	**<0.01**
**Adjusted satisfaction score for care by nurses/nurses' aides, mean (SD)**	6734	0	81.1 (1.8)	80.9 (1.8)	**<0.01**
**Adjusted satisfaction score for care by physicians/surgeons, mean (SD)**	6734	0	79.7 (2)	79.6 (2.1)	0.41
**Donor coordinating team staff**					
**Turnover of physicians, n (%)**					
No	4944 (73)	0	3495 (70.7)	1449 (29.3)	0.43
Yes	1790 (27)		1283 (71.7)	507 (28.3)	
**Proportion of new DCT nurses, mean (SD)**	6734	0	14 (15.8)	15 (16.1)	**0.02**
**Ratio FTE DCT physicians/100 potential donors, n (%)**					
<3	5747 (85)	0	4047 (70.4)	1700 (29.6)	**0.02**
≥3	987 (15)		731 (74.1)	256 (25.9)	
**Ratio FTE DCT nurses/100 potential donors, (%)**					
<8	2701 (40)	0	1852 (68.6)	849 (31.4)	**<0.01**
≥8	4033 (60)		2926 (72.6)	1107 (27.4)	
**Difference FTE physicians recommended/observed, n (%)**	6734		0.2 (0.4)	0.2 (0.4)	**<0.01**
**Difference FTE DCT nurses recommended/observed, n (%)**	6734		0.5 (0.7)	0.6 (0.8)	0.84
**Mean number of after-hours offsite on-call duty shifts, n (%)**					
None	3880 (58)	0	2708 (69.8)	1172 (30.2)	**0.04**
<2	1178 (17)		847 (71.9)	331 (28.1)	
≥2	1676 (25)		1223 (73)	453 (27)	

UHC: university hospital centre; ABM: Agence de la biomédecine; HAS: Haute autorité de santé; FTE: full-time equivalent: DCT: donor coordination team.

Table S2. Multilevel logistic regression models of factors related to refusal of organ donation.

	Univariate Model			Step 2 Model			Step 3 Final Model		
	OR	95% CI	*P* value	aOR	95% CI	*P*-value	aOR	95% CI	p-value
Donor factors									
**Donor age, years**			<0.0001			<0.0001			<0.0001
0 to 17	2.17	1.59 to 2.95		2.18	1.60 to 2.97		**2.10**	**1.54 to 2.86**	
18 to 49	1.54	1.34 to 1.77		1.58	1.37 to 1.83		**1.58**	**1.37 to 1.83**	
50 to 64	1.36	1.19 to 1.56		1.37	1.20 to 1.57		**1.38**	**1.20 to 1.58**	
≥65	1	-		1	-		1	-	
**Donor sex**			0.21						
Female	1	-							
Male	1.07	0.96 to 1.20							
**Blood group**			0.01			0.01			0.02
A	0.98	0.87 to 1.11		0.98	0.87 to 1.10		0.99	0.87 to 1.11	
AB	1.08	0.82 to 1.43		1.07	0.81 to 1.41		1.07	0.81 to 1.42	
B	1.36	1.13 to 1.63		1.33	1.10 to 1.59		**1.32**	**1.10 to 1.59**	
O	1	-		1	-		1	-	
**Cause of death**			0.01						
Vascular	1	-							
Trauma	1.05	0.91 to 1.21							
Anoxia	1.29	1.11 to 1.49							
Other	1.28	0.91 to 1.79							
**Suicide**			0.21			0.01			0.01
No	1	-		1	-		1	-	
Yes	0.86	0.68 to 1.09		0.73	0.58 to 0.93		**0.74**	**0.58 to 0.93**	
Hospital-related factors									
**Hospital in overseas region or territory**			0.47						
No	1	-							
Yes	0.84	0.52 to 1.35							
**Type of hospital**			<0.01						
UHC	1	-							
Non-UHC	0.75	0.61 to 0.91							
**Number of beds of hospital site of death**			0.80						
<200	1.01	0.70 to 1.46							
200 to 299	0.92	0.69 to 1.22							
300 to 399	0.86	0.64 to 1.16							
400 to 499	0.86	0.63 to 1.18							
≥500	1	-							

**Neurovascular unit**			0.64			
No	1	-				
Yes	1.06	0.84 to 1.32				
**Hospital audited by the ABM**			0.08			0.02
No	1	-		1	-	
Yes	0.79	0.60 to 1.03		**0.74**	**0.58 to 0.95**	
HAS quality indicators						
**Level of certification**			0.59			
Certification with or without recommendations for improvement	1	-				
Certification with obligations to improve or decision suspended	0.91	0.64 to 1.28				
**Adjusted satisfaction score for reception**	0.98	0.95 to 1.00	0.09			
**Adjusted outpatient satisfaction score**	0.94	0.90 to 0.98	0.01			
**Adjusted inpatient satisfaction score**	0.95	0.91 to 0.99	0.01	**0.95**	**0.92 to 0.99**	0.01
**Adjusted satisfaction score for care by nurses/nurses' aides**	0.94	0.89 to 0.99	0.02			
**Adjusted satisfaction score for care by physicians/surgeons**	0.97	0.92 to 1.01	0.16			
Donor coordinating team						
**Turnover of physicians**			0.80			
No	1	-				
Yes	0.97	0.77 to 1.23				
**Mean proportion of new DCT nurses**	1	1.00 to 1.01	0.17			
**Ratio of FTE DCT physicians/potential donors**			0.14			
<3 FTE DCT physicians per 100 potential donors	1	-				
≥3 FTE DCT physicians per 100 potential donors	0.83	0.65 to 1.06				
**Ratio of FTE DCT nurses/potential donors**			<0.01			0.01
<8 FTE DCT nurses per 100 potential donors	1	-		1	1	
≥8 FTE DCT nurses per 100 potential donors	0.74	0.60 to 0.92		**0.78**	**0.64 to 0.95**	
**Difference in FTE DCT physicians recommended/observed**	1.43	1.07 to 1.90	0.01			
**Difference in FTE DCT nurses recommended/observed**	1.01	0.88 to 1.17	0.84			
**Mean number of after-hours offsite on-call duty shifts**			0.10			
None	1	-				
<2	0.99	0.76 to 1.30				
≥2	0.78	0.62 to 0.98				

Step 2 model: multivariate model—donor-related factors. Step 3 final model: multivariate model—donor- and hospital-related factors. OR: odds ratio; aOR: adjusted odds ratio; 95% CI: 95% confidence interval; ABM: Agence de la biomédecine; HAS: Haute autorité de santé (National Authority for Health); UHC: university hospital center; FTE: full-time equivalent: DCT: donor coordination team .
